# The Effect of NSAID Pretreatment on Aqueous Humor Prostaglandin E_2_ Concentration in Eyes Undergoing Femtosecond Laser-Assisted Capsulotomy

**DOI:** 10.1155/2018/1891249

**Published:** 2018-07-10

**Authors:** Vasilios F. Diakonis, Apostolos G. Anagnostopoulos, Angeliki Moutsiopoulou, Nilufer Yesilirmak, Florence Cabot, Daniel P. Waren, Terrence P. O'Brien, Sonia H. Yoo, Robert J. Weinstock, Kendall E. Donaldson

**Affiliations:** ^1^Bascom Palmer Eye Institute, University of Miami Miller School of Medicine, Miami, FL, USA; ^2^Department of Chemistry, University of Miami, Miami, FL, USA; ^3^The Eye Institute of West Florida, Largo, FL, USA

## Abstract

**Purpose:**

To assess aqueous humor concentration of prostaglandin E_2_ (PGE_2_) after capsulotomy creation using a femtosecond laser (FLAC) in patients pretreated with short-term topical ketorolac versus patients without pretreatment.

**Methods:**

This prospective study comprised consecutive patients scheduled to undergo cataract surgery using a femtosecond laser platform to perform only capsulotomies. An identical protocol for preoperative mydriasis was used for all the eyes included in the study, while aqueous humor was extracted from the anterior chamber of all patients immediately after the initial side port incision. ELISA was performed to quantify aqueous humor PGE_2_. The patients were divided into 2 groups; in group 1, the patients received short-term topical ketorolac preoperatively, while the patients in group 2 did not receive NSAID pretreatment.

**Results:**

Twenty eyes of 20 patients were included in the study (10 eyes in each group). Mean concentration of aqueous humor PGE_2_ after FLAC was 392.16 ± 162.00 pg/ml and 622.63 ± 331.84 pg/ml for groups 1 and 2, respectively. A statistically significant difference in aqueous humor PGE_2_ concentration between the two groups (*p* < 0.05) was demonstrated, with the eyes that received ketorolac pretreatment demonstrating a lower concentration of PGE_2_.

**Conclusion:**

Short-term topical use of ketorolac prior to FLAC seems to prevent excessive release of PGE_2_ in the anterior chamber of the eyes that received NSAID pretreatment when compared to the eyes that did not receive NSAIDs preoperatively.

## 1. Introduction

Cataract surgery requires sufficient mydriasis, to facilitate capsulorhexis or capsulotomy, phacoemulsification, and finally intraocular lens insertion. Even though all patients undergoing cataract surgery receive preoperatively topical mydriatic agents, intraoperative pupillary miosis may occur due to the release of inflammatory mediators (prostaglandins) or due to iris surgical trauma [1] Miotic pupils during cataract surgery have been associated with increased complication rates during cataract surgery, leading in some cases in visual loss [2, 3].

The pretreatment with femtosecond laser-assisted cataract surgery (FLACS) platforms during cataract surgery has been associated with pupillary miosis [4–6]; the latter has been attributed to the increase of inflammatory mediator (prostaglandins) concentration in the anterior chamber (AC) after FLACS [7, 8]. Recent studies have demonstrated that short-term topical NSAIDs as a pretreatment prior to FLACS results in less prostaglandin release in the AC [9], and also NSAID pretreatment seems to induce significantly less pupillary miosis when compared to the eyes that did not receive NSAID prior to FLACS [10, 11]. The patients in the study assessing prostaglandin quantification in the AC received both capsulotomy and lens fragmentation during FLACS [9], while the NSAID used was diclofenac [9].

The following study will assess aqueous humor prostaglandin E2 (PGE_2_) concentrations after FLAC (lens fragmentation may influence the inflammatory response and hence prostaglandin production due to cataract density disparities between the eyes) in patients who were pretreated with topical ketorolac compared with patients who did not receive NSAID pretreatment.

## 2. Methods

### 2.1. Patient Population

This prospective, randomized, observational case series included 20 consecutive patients (one eye was included per patient, 14 were male and 16 female, aged 68.34 ± 8.65 years (range, 50–90 years)) undergoing cataract surgery using FLACS between July 2015 and July 2016. FLACS pretreatment included the creation of only the capsulotomy, using the Catalys (Abbott Medical Optics Inc., Santa Ana, California, USA) laser platform. The interface utilized in all cases was the Liquid Optics Interface, 14.0 mm inner diameter (Abbott Medical Optics Inc., Santa Ana, California, USA). An identical protocol for preoperative mydriasis was used for all the eyes, while pupil diameter was evaluated before FLACS. The patients were divided into 2 groups; in group 1 (10 eyes) the patients received short-term topical NSAIDs preoperatively (ketorolac, 4 times per day for 3 days prior to cataract extraction), while the patients in group 2 (10 eyes) did not receive NSAID pretreatment. Furthermore, intraoperative grading of capsulotomy creation was performed as follows: type 1 (complete treatment pattern or free floating), type 2 (microadhesions), type 3 (incomplete treatment pattern), and type 4 (complete pattern, but not continuous), as described by a previous study [11].

All patients were informed of the risks and benefits prior to cataract surgery, and they gave written informed consent in accordance with institutional guidelines and the Declaration of Helsinki for human research. Prior to the study, an institutional review board approval was obtained.

### 2.2. Inclusion and Exclusion Criteria

Patients included in the study had an unremarkable ocular history. Glaucoma patients receiving topical treatment or patients with any other ocular or systemic disease involving the eyes such as inflammatory eye disease, pseudoexfoliation, diabetes mellitus, history of treatment with an *α*-adrenergic-antagonist, history of poor pupillary dilation (less than 6.0 mm), previous ocular surgery or trauma, or rheumatic disease were excluded from the study.

### 2.3. Cataract Surgery Technique

Preoperative mydriasis was performed using topical 1.0% tropicamide (Alcon Inc., Lake Forest, IL, USA) eye drops and 2.5% phenylephrine (Paragon BioTeck Inc., Portland, OR, USA) eye drops, instilled three times (every 10 minutes) within 1 hour prior to FLACS (the last application of topical mydriatics was instilled 15 minutes prior to surgery). All procedures were performed under topical anesthesia; a 5.0 mm in diameter femtosecond capsulotomy was performed in all cases, while corneal incisions and lens fragmentation was not performed in any case. Clear corneal incisions were performed manually, followed by standard phacoemulsification (Centurion, Alcon, Fort Worth, TX, USA). The capsulotomy settings were identical for all cases: the pattern was circular with a 5.0 mm diameter centered at the pupil and the incision depth was 600 microns. The horizontal and vertical spot spacings were 5 and 10 microns, respectively, while the pulse energy was 4 microjoules.

Five minutes after capsulotomy creation and prior to traditional phacoemulsification, a 1.15 mm paracentesis port was made, and aqueous humor was collected in a syringe (a volume of 100 microliters or more was considered to be an adequate sample); all aqueous humor samples were immediately stored in −80°C and were only again in room temperature conditions prior to performing ELISA. No intraoperative complications were noted in any of the cases included in the study, while an intraocular lens was placed in the capsular bag in all cases.

Postoperatively, all patients received the same treatment regimen: a combination of an antibiotic, steroid, and nonsteroidal anti-inflammatory agent.

### 2.4. ELISA Assessment

All aqueous humor samples from both groups were assessed by the same investigator with the same standard preparation to avoid interuser or plate variability. PGE_2_ concentrations were determined using a commercially available PGE_2_ Parameter Assay Kit (R&D Systems, Minneapolis, MN) according to the manufacturer's instructions. Measurements were performed using a microplate reader (Clariostar Monochromator Microplate Reader, BMG LABTECH, Ortenberg, Germany).

### 2.5. Statistical Analysis

Comparisons between groups were performed using Student's *t*-test. Statistical analysis was performed using the SPSS statistical package (version 22; IBM Software). A *P* value of 0.05 or less was considered statistically significant in our analyses.

## 3. Results

Twenty eyes (10 eyes per group) of 20 patients were included in the study ((20 patients, one eye was included per patient, 14 were male and 16 female, aged 68.34 ± 8.65 years (range, 50–90 years)). There was no statistically significant difference between the 2 groups in terms of age (*p* > 0.05). All femtosecond laser-assisted capsulotomies in both groups were graded as type 1 (complete capsulotomies or free floating).

Mean concentration of aqueous humor PGE_2_ after FLAC was 392.16 ± 162.00 pg/ml (range: from 202.04 to 713.71 pg/ml) and 622.63 ± 331.84 pg/ml (range: from 310.09 to 1469.74 pg/ml) for groups 1 and 2, respectively. There was a statistically significant difference in aqueous humor PGE_2_ concentration between the two groups (*p*=0.03), with the eyes that received ketorolac pretreatment demonstrating lower concentrations of PGE_2_.

## 4. Discussion

Previous studies have shown that FLACS pretreatment results in significant pupillary miosis [4–6]. A published study from our department that evaluated the effect of 3 femtosecond laser platforms after FLACS pretreatment on pupil diameter also revealed significant pupillary miosis which was independent to the laser platform used [6], It is hypothesized that pupillary miosis is associated with prostaglandin release in the AC and with the dissipation of laser energy around the iris tissue [5, 6]. In support of the above theories, a study by Schultz et al. [7] reported significant increase of prostaglandins in the aqueous humor after FLACS. Furthermore, a study by Jong et al. [8] associated pupillary miosis with the with the patient's age [8] and the total time of laser application, while suction time (of the patient interface) did not seem to effect pupil diameter [8].

The use of topical NSAIDs prior to cataract surgery has been regularly used as the standard of care to minimize pupillary miosis during surgery since the mid 1980s. It seems that the intraocular manipulations during cataract surgery cause breakdown of the blood-aqueous barrier promoting an inflammatory cascade with the release of prostaglandins by ciliary body [12–15]. The topical use of NSAIDs inhibits the synthesis of prostaglandins in the AC and thereby minimizes the likelihood of pupillary miosis [16–18]. A recent study from our institute demonstrated that patients receiving a 3-day topical regimen of ketorolac prior to FLAC better retained their mydriasis after the laser treatment [10]. Furthermore, a study by Schultz et al. showed that the use of topical NSAIDs (diclofenac) prior to FLACS reduced prostaglandin release in the AC [9]. In the study mentioned above, the authors performed FLACS treatment that included capsulotomy creation and lens fragmentation, while diclofenac was used as an NSAID agent treatment at the day of surgery [9], while in our previous study, only FLACS capsulotomy was performed (to better control the study parameters) and ketorolac was used prior to surgery [10].

The current study evaluated aqueous humor concentration of PGE_2_ after FLAC (only capsulotomy was performed), using the same laser platform and the same settings for capsulotomy creation in all eyes included. This study approach was designed to control the effects of laser energy dissipation in the AC (only femtosecond capsulotomy which seems to be the main inducer of prostaglandin release [19]) avoiding the possible implications that cataract density disparities that could influence prostaglandin release (increase the variables of the current study). Furthermore, ketorolac was used and its topical application commenced 3 days prior to cataract surgery (4 times per day) in contrast to the Schultz et al. study where diclofenac was used one day prior to cataract surgery; a recent study by Kiss et al. utilized nepafenac one day prior to FLACS and demonstrated significant reduction of prostaglandin concentration in the aqueous humor when compared even with manual cataract surgery [20]. Our study demonstrated that short-term (3 days prior to FLAC, we utilized 3 days of NSAID as we followed the standard protocol used in our department for the preoperative regimen, and as mentioned above, other studies demonstrate that the shorter preoperative regimen seems equally efficacious to ours) topical use of ketorolac prior to FLAC seems to prevent excessive release of PGE_2_ in the anterior chamber of the eyes that received NSAID pretreatment when compared to the eyes that did not receive NSAIDs. These findings were similar to the published study by Schultz et al.; these two studies suggest that any FLACS treatment may lead to increased PGE_2_ levels in the AC and subsequently to pupil miosis. Finally, the use of different NSAID agents either immediately prior to cataract surgery or for a few days prior to surgery seems to well control the release of PGE_2_ in the AC.

Our findings reveal that even capsulotomy creation which secures short laser time exposure, minimal laser energy utilization, and short suction time by the patient interface in patients with no comorbidities may cause a significant increase in PGE_2_ levels in the AC. Furthermore, a 3-day pretreatment with topical ketorolac prior to FLACS seems to significantly inhibit FLACS-induced PGE_2_ release in the AC when compared with patients who did not receive a NSAID pretreatment. There was a difference between the findings of this study and the study by Schultz et al. in terms of the actual concentration values of PGE_2_, with our study demonstrating much higher concentrations in both the NSAID and no NSAID group. This may be attributed to the ELISA kit used which could have different sensitivity; furthermore, this could be a result of different dilution the aqueous humor samples underwent in these two studies prior to ELISA. Finally, the concentration differences quantified by different ELISA kits could be attributed to their specificity and more specifically to the cross-reactivity they demonstrate.

The current study is limited by the small sample of eyes included and the fact that we did not include more groups to assess other NSAID agents. Despite the above limitations, we demonstrate that a 3-day (4 times per day) topical use of an NSAID agent (ketorolac) prior to FLAC seems to prevent excessive release of PGE_2_ in the anterior chamber. Additionally, the eyes that did not receive topical NSAID pretreatment demonstrated higher concentrations of prostaglandins on the AC when compared with the eyes that received the NSAID prior to FLACS that could potentially lead to significant pupillary miosis. It is suggested that patients undergoing any type of FLACS treatment prior to cataract surgery to receive a course of topical NSAID regimen to avoid pupillary miosis and miosis-related complications.
